# Moraxella catarrhalis Septic Arthritis Unveils Undiagnosed Systemic Lupus Erythematous in a Pediatric Patient

**DOI:** 10.7759/cureus.50909

**Published:** 2023-12-21

**Authors:** Nathaniel G Rogers

**Affiliations:** 1 Department of Internal Medicine and Pediatrics, The University of Tennessee Health Science Center, Memphis, USA

**Keywords:** monoarticular, elbow, systemic lupus erythematosis, moraxella, pediatrics, septic arthritis

## Abstract

Septic arthritis is uncommon in pediatric patients, who are less likely to have major risk factors such as underlying joint disease or prosthetic joints. It only rarely affects the elbow and is usually caused by Gram-positive cocci, with *Staphylococcus aureus* being the most common bacterial organism. We present the case of a 15-year-old previously healthy female who experienced new-onset monoarticular nontraumatic elbow pain and was found to have a synovial effusion growing from *Moraxella catarrhalis*. The atypical clinical presentation, coupled with the growth of an unusual organism, raised concern for an underlying immunocompromising or inflammatory joint disorder. Further laboratory workup ultimately revealed a diagnosis of systemic lupus erythematosus (SLE), which more commonly presents with arthralgias that are polyarticular, symmetric, and migratory. This case report should encourage clinicians to maintain a high degree of suspicion for underlying joint disease when septic arthritis presents atypically.

## Introduction

Septic arthritis is an uncommon disease in children, affecting approximately 8 cases per 100,000 patients per year, with a higher prevalence in those five years old and younger. Hips, knees, and ankles are the most commonly affected joints in children [1]. The pathophysiology of septic arthritis generally occurs via hematogenous spread, extension of soft tissue infection into a nearby joint, or direct inoculation after an injury [1]. Risk factors for septic arthritis in children with sickle cell disease include a lack of full immunizations, recent pharyngitis, exposure to animals, and recent diarrheal illness [1]. Septic arthritis management generally involves prompt consultation with orthopedic surgery and empiric antibiotics once blood and synovial cultures have been drawn [2,3].

Systemic lupus erythematosus (SLE) is a systemic autoimmune disease with variable clinical manifestations. It mainly affects females and can present with various clinical signs, symptoms, and laboratory workups [4]. Its diagnosis is clinical, using expert opinion classification criteria such as the 1997 American College of Rheumatology Criteria and the 2012 Systemic Lupus International Collaborating Clinics or the 2019 European Alliance of Associations for Rheumatology/ACR criteria. All these classifications take into account physical exam findings such as rash, arthritis, and fever, as well as laboratory workups such as proteinuria, auto-antibodies, and complement levels. While arthritis and arthralgias occur in a vast majority of patients with SLE, they tend to be migratory, polyarticular, and symmetrical [5,6]. Thus, monoarticular arthritis is unusual and typically points away from the diagnosis of SLE [7].

While patients with underlying inflammatory joint disorders are at higher risk of developing joint infections, septic arthritis is still an uncommon presenting finding for the diagnosis of SLE [8]. The presentation of septic arthritis can range from being clinically apparent to having various non-specific findings, thus requiring a physician to have a high index of clinical suspicion. A delayed diagnosis may lead to irreversible damage to the involved joint [9]. The most common causative organism in septic arthritis is Staphylococcus aureus. S. aureus was found to be the causative organism in 76% of cases in a specific retrospective review in children, followed by various streptococcal organisms (approximately 16%); Gram-negative infection was reported in just 8% to 14% of cases [10]. Additionally, despite medical therapies for SLE having advanced in recent years, infection is one of the more common reasons for mortality, especially once the patient develops bacteremia. Joint infections have been cited to cause anywhere between 7% and 15% of mortality for patients with SLE [10-13].

## Case presentation

A 15-year-old previously healthy female presented with two days of right elbow pain and swelling without any history of trauma. Other than occasional headaches, she denied systemic symptoms such as weight loss, fever, recent illnesses such as pharyngitis, urinary tract infections, pneumonia, or diarrhea, as well as a recent history of travel or steroid use. Given her increasing pain to the point where she was unable to actively mobilize her elbow, her caregiver brought her to the emergency department for evaluation. An ultrasound was obtained showing a joint effusion, for which orthopedic surgery was consulted, and an arthrocentesis was performed. Magnetic resonance imaging had no findings suggestive of an underlying fracture or recent hemorrhagic effusion, which could have been suggestive of recent trauma, and excluded osteomyelitis. The patient was admitted for observation and the collection of blood and synovial fluid cultures without the initiation of antibiotics. That evening, the synovial fluid results demonstrated an elevated white blood cell count (WBC) of 18,500 per microliter with 80% neutrophils and a negative gram stain. Additionally, the patient became febrile, and thus vancomycin and ceftriaxone were empirically started as concerns for septic arthritis.

Her synovial culture grew Moraxella catarrhalis, which prompted an infectious diseases consultation. Further workup with testing of HIV, rapid plasma reagin, gonorrhea, and chlamydia were all negative. Blood cultures remained negative. Laboratory investigation demonstrated leukopenia and neutropenia with a total serum WBC of 2,000 per microliter and an absolute neutrophil count of 1100 per microliter. An auto-immune workup was initiated due to this atypical cause of septic arthritis and leukopenia. Her laboratory values, as shown in Table 1, were noteworthy for low complement levels, elevated inflammatory markers, positive anti-nuclear antibody (ANA), direct agglutinin test (DAT) positive anemia, and a strongly positive double-stranded DNA antibody (ds-DNA). According to the 2012 Systemic Lupus Erythematosus International Collaborating Clinics (SLICC) criteria, this presentation met the criteria for a new diagnosis of SLE [14].

**Table 1:** Patient hematologic laboratory values upon admission ab: antibody, fL: femtoliters, mcL: microliters, mL: milliliter, dL: deciliter, L: liters, mm: millimeters, mg: milligrams, g: grams, IU: international units, EU: endotoxin unit, I: index. *Double-stranded DNA antibody parameters: <25.0 IU/ml negative, 25.0-29.99 IU/ml equivocal, 30.0–59.99 IU/ml weakly positive, 60.0-200.0 IU/ml moderately positive, >200.0 IU/ml strongly positive.

Hematologic test	Patient value	Reference value
White blood cells (thousands/mcL)	2.0	5.0–11.0
Hemoglobin (g/dL)	10.9	12.0–16.0
Hematocrit (%)	32.7	36.0–48.0
Mean corpuscular volume (fL)	74.5	78.0–98.0
Platelets (thousand/mcL)	152	140–450
Neutrophils (in %)	54.4	40–60
Absolute neutrophil count (thousand/mcL)	1.1	>1.8
Erythrocyte sedimentation rate (mm/hour)	83	0–13
C-reactive protein (mg/L)	23.9	<9
Anti-nuclear antibody index (I)	>5.6	<1.09
Double-stranded DNA ab (IU/mL)*	>320	<30.0
C3 complement (mg/dL)	27.0	90.0–180.0
C4 complement (mg/dL)	3.4	10.0–40.0
Anti-Smith ab (EU/mL)	1.7	0–15.9
Anti-RNP ab (EU/mL)	1.1	0–15.9
Anti-SSA ab (EU/mL)	7.4	0–15.9
Anti-SSB ab (EU/mL)	2.5	0–15.9
Rheumatoid factor (IU/mL)	<10	0–15
Direct agglutinin test	Positive	Negative

Given her active bacterial infection, no steroids were started at the time of diagnosis. During her hospital course, she developed an acute kidney injury, which, in conjunction with her new diagnosis of SLE, led to a kidney biopsy showing class I lupus nephritis, further confirming the diagnosis. After completion of ten days of intravenous ceftriaxone for septic arthritis, the patient was started on 2 mg/kg per day of IV methylprednisolone with improvement of renal function and leukopenia. She was discharged with amoxicillin/clavulanic acid (a total of 21 days of therapy), oral steroids, and hydroxychloroquine, with a rheumatology follow-up.

## Discussion

This case of a 15-year-old female who presented with new-onset monoarticular nontraumatic joint pain underscores the importance of having a high index of suspicion for underlying immunocompromise when an uncommon organism causes septic arthritis. Several clinical clues pointed the physician team towards an underlying joint disorder. First, the greatest risk factors for septic arthritis are having a non-native joint or pre-existing joint disease, which is found in over half of patients [15]. Second, the elbow is an uncommon location for a joint infection in children, especially without direct inoculation or injury to the joint, accounting only for 8% of septic arthritis cases [10]. Third, when synovial fluid cultures grew the unusual organism Moraxella catarrhalis, concern was heightened for underlying immunocompromise or an inflammatory joint disorder. Fortunately, M. catarrhalis typically responds well to timely antibiotic therapy, and in this case, rapid identification and initiation of treatment prevented permanent joint damage [16].

A review of the literature reveals that Moraxella is an uncommon cause of septic arthritis. One study of 505 cases of septic arthritis in both adults and children over 10 years did not report a single case of Moraxella as the causative agent [17]. In case reports of Moraxella septic arthritis, the patients often had a known underlying comorbidity that made them susceptible. Cases included patients with a prosthetic knee joint [18], history of stem cell transplant [16], pregnancy with concurrent HIV and intravenous drug use [19], multiple myeloma [20], post-traumatic injury [21], hemodialysis treatment [21], or immunocompromise after recent treatment with infliximab [22]. Of note, one other case report did show culture-proven Moraxella septic arthritis in a patient with SLE; however, she had recently completed a prednisone taper for an acute lupus flare [23]. Only rarely has the Moraxella genus of bacteria in septic joints been isolated from immunocompetent adults and children [24-26]. In some cases, Moraxella could not be detected on culture and had to be detected via PCR. Thus, clinicians must be aware of this possibility and use their clinical index of suspicion to guide further testing [24,27].

## Conclusions

This case is unique in multiple regards: our previously healthy pediatric patient presented with septic arthritis in an uncommon joint caused by an unusual organism, Moraxella catarrhalis, leading to a new diagnosis of systemic lupus erythematosus. This case presentation should encourage clinicians to maintain a high degree of suspicion for underlying joint disease when septic arthritis presents atypically.
